# Dopant‐Free and Carrier‐Selective Heterocontacts for Silicon Solar Cells: Recent Advances and Perspectives

**DOI:** 10.1002/advs.201700547

**Published:** 2017-12-04

**Authors:** Pingqi Gao, Zhenhai Yang, Jian He, Jing Yu, Peipei Liu, Juye Zhu, Ziyi Ge, Jichun Ye

**Affiliations:** ^1^ Ningbo Institute of Material Technology and Engineering Chinese Academy of Sciences Ningbo 315201 China; ^2^ University of Chinese Academy of Sciences Beijing 100049 China

**Keywords:** carrier‐selective contacts, dopant‐free, heterojunction solar cells, poly(3,4‐ethylene dioxythiophene):poly(styrenesulfonate) (PEDOT:PSS), transition metal oxides

## Abstract

By combining the most successful heterojunctions (HJ) with interdigitated back contacts, crystalline silicon (c‐Si) solar cells (SCs) have recently demonstrated a record efficiency of 26.6%. However, such SCs still introduce optical/electrical losses and technological issues due to parasitic absorption/Auger recombination inherent to the doped films and the complex process of integrating discrete p^+^‐ and n^+^‐HJ contacts. These issues have motivated the search for alternative new functional materials and simplified deposition technologies, whereby carrier‐selective contacts (CSCs) can be formed directly with c‐Si substrates, and thereafter form IBC cells, via a dopant‐free method. Screening and modifying CSC materials in a wider context is beneficial for building dopant‐free HJ contacts with better performance, shedding new light on the relatively mature Si photovoltaic field. In this review, a significant number of achievements in two representative dopant‐free hole‐selective CSCs, i.e*.*, poly(3,4‐ethylene dioxythiophene):poly(styrenesulfonate)/Si and transition metal oxides/Si, have been systemically presented and surveyed. The focus herein is on the latest advances in hole‐selective materials modification, interfacial passivation, contact resistivity, light‐trapping structure and device architecture design, etc. By analyzing the structure–property relationships of hole‐selective materials and assessing their electrical transport properties, promising functional materials as well as important design concepts for such CSCs toward high‐performance SCs have been highlighted.

## 1 Introduction

In the last few decades, the photovoltaic (PV) market has been dominated by crystalline silicon (c‐Si) solar cells (SCs) due to their overwhelming advantages in efficiency, cost, stability, and security. Recently, a record efficiency of 26.6% has been achieved combining the most successful heterojunctions (HJs) with interdigitated back contacts (IBCs).^1^ The IBC design is straightforwardly beneficial to light harvesting and passivation at the front side of the cell in comparison with the traditional double‐sided junctions. Another main reason for this efficiency progress can be ascribed to the ‘passivating contact' or ‘carrier‐selective contacts' (CSCs) technology enabled by HJs, which use intrinsic amorphous silicon (a‐Si) to passivate the Si surface and doped a‐Si to delivery hole/electron‐selective transport. Relying on the nearly ideal asymmetric band offset design at the CSC regions, the photoexcited electrons and holes can be efficiently separated and collected toward the opposite electrodes, and so, the devices with the HJ‐IBC design have extremely high open‐circuit voltages (*V*_oc_) and efficiencies. However, such SCs still introduce optical/electrical losses and technological issues due to parasitic absorption/Auger recombination inherent to the doped a‐Si films and complex processing in integrating discrete p^+^ and n^+^ HJ contacts to the back side of cell.^2^ These issues have motivated research into searching for alternative new functional materials as well as simplified deposition technologies whereby CSCs with c‐Si substrates, and thereafter IBC cells, can be formed directly via a dopant‐free manner.

Actually, the concept of CSCs, which was previously explored for organic devices, has now been paid much interest in perovskite as well as c‐Si SCs. From the energy band alignment views, the well‐known transition metal oxides (TMOs) (molybdenum oxide (MoO*_x_*), vanadium oxide (V_2_O*_x_*), tungsten oxide (WO*_x_*), etc.),^3^ poly(3,4‐ethylene dioxythiophene):poly(styrenesulfonate) (PEDOT:PSS),^4^ graphene,^5^ carbon nanotubes,^6^ etc., all with high work functions (WFs) and hole conducting properties, can act as hole‐selective layers (HTLs), while the low WF metals^7^, ^8^ or metal oxides (MOs),^9^, ^10^, ^11^, ^12^ alkali/alkaline earth metal salts,^13^, ^14^ and some organic materials^15^, ^16^ can serve as electron‐selective layers (ETLs) via well‐matched conduction band alignment. In the past five years, many of these contact systems have already been successfully implemented on the c‐Si absorbers and have demonstrated promising efficiencies of 19.4% and 22.5%, respectively, for the heterojunction solar cells (HSCs) featuring either full or half dopant‐free heterocontacts.^17^, ^18^ With the abovementioned materials and new CSC materials that emerge constantly, great advances are made in the scope and depth of research in dopant‐free c‐Si heterocontacts. It is believed that screening and modifying CSC materials in a wider context is beneficial for building dopant‐free HJ contacts with even better performance, shedding new light on the relatively mature Si PV techniques. Moreover, most HTL and ETL materials can be deposited via low temperature and/or solution‐based processing, such as spin‐coating or thermal evaporation,^19^, ^20^ providing big potentials in both cost reduction and procedure simplification (especially for IBC). The carrier‐selective and dopant‐free contacts are therefore of great interest to not only the fundamental researchers but also the PV industry, and a timely and systematic review would be very helpful in summarizing the fragmentary subjects and refining the research emphases.

In this review, a significant number of achievements in two representative dopant‐free HTL CSCs, i.e., PEDOT:PSS/Si and TMO/Si, will be systemically presented and surveyed. Focuses will be placed on the latest advances in the modification of HTL materials, interfacial passivation, contact resistivity, design of light‐trapping and device structures, etc. By analyzing the structure–property relationships of HTL materials and assessing their electrical transport properties, the promising HTL materials as well as the important design concepts for such CSCs toward high performance SCs will be highlighted. Finally, the perspectives and challenges in the research area of dopant‐free and carrier‐selective contacts will be presented in combination with the development of easily fabricated IBC c‐Si SCs. Because of a limit on the paper length, this review lacks the survey and analysis of ETL materials and electron‐selective contacts.

## 2 PEDOT:PSS/Si HSCs

**Figure 1:**
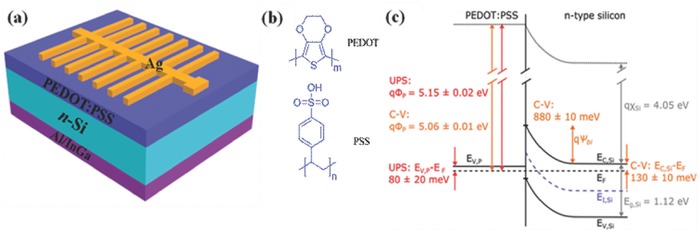
a) Schematic diagram of PEDOT:PSS/n‐Si HSCs; b) chemical structural formulae of the PEDOT and PSS; c) junction formation at the hybrid PEDOT:PSS/n‐Si interface. Reproduced with permission.^4^ Copyright 2015, Nature Publishing Group.

Initial research on the PEDOT:PSS/Si HSCs came from Jabbour and co‐workers who used an organic PEDOT:PSS layer to replace the boron‐doped amorphous silicon carbide (a‐SiC:H) layer in microcrystalline silicon SCs and achieved a relatively high *V*_oc_ of 0.88 V.^21^ Though the incipient efficiency (≈2.1%) was not satisfactory in comparison with traditionally diffused c‐Si homojunction SCs, the potential development of this kind of SCs still attracts wide attention due to their dopant‐free concept as well as their vacuum‐free, low‐temperature and solution‐based fabrication procedures. The concept was then transferred to n‐type c‐Si (n‐Si) substrates. The device configuration of PEDOT:PSS/n‐Si HSCs and the chemical structures of PEDOT and PSS are presented in **Figure** **1**a,b.^22^, ^23^ The device fabrication can be implemented by spin‐coating PEDOT:PSS on top of the n‐Si substrate and then depositing the front and rear electrodes. In this kind of CSC, the HTL layer of PEDOT:PSS plays multiple roles, including antireflection coating layer (ARC), charge extraction layer, electron‐blocking/hole‐transporting layer, surface passivation layer, etc. Therefore, the PEDOT:PSS layer shall satisfy the all‐around requirements in conductivity and WF as well as the contact property with the Si surface in order to achieve high‐performance SCs. Through extensive optimization of the photoelectric property of the PEDOT:PSS film, PEDOT:PSS/n‐Si hetero‐interface and n‐Si/rear electrode contact, the HSCs have achieved an efficiency over 16%.^24^, ^25^

### 2.1 Transport Mechanism

For the PEDOT:PSS/n‐Si HSCs, highly doped PEDOT:PSS with a WF of 5.0–5.1 eV is usually treated as a quasi‐metal or a p‐type semiconductor. Once contact is established between the PEDOT:PSS and the n‐Si substrate, an inversion layer is introduced near the Si surface due to the large conduction band offset that stems from the difference between the Fermi level of n‐Si and the WF of PEDOT:PSS.^4^, ^22^, ^26^, ^27^, ^28^, ^29^ As shown in Figure 1c, this conduction band offset at the depletion region provides an effective barrier for blocking electrons moving from the bulk Si to the PEDOT:PSS layer, while the favorable valence band offset contributes to effective holes' transport. The inversion effect is thus rationally interpreted as a driving force that promotes the hybrid device similar to a common p^+^n junction. Theoretically, this inversion layer enables a sufficiently high built‐in potential of 880 meV seen from the Si side.

A comprehensive theory based on the p^+^n heterojunction model was presented for the first time by Jäckle et al.^4^ They clearly showed that the hybrid heterojunction of PEDOT:PSS with n‐Si behaves similarly to a conventional p–n junction, in which the transport is governed by the diffusion of minority charge carriers in the n‐Si. In contrast to the Schottky junction, the dark saturation current density (*J*_0_) for a one‐sided abrupt p^+^n junction can be defined by Equation (1)

(1) $$J_{0}=n_{i}{}^{2}\mu_{p}KT/L_{p}N_{D}$$

where *n*_i_ is the intrinsic carrier concentration, μ_p_ is the mobility of holes, *L*_p_ is the diffusion length of the minority carriers (holes for n‐Si), and *N*_D_ is the doping concentration of an n‐Si substrate. The p^+^n junction model was further supported by experimental results, as shown in **Table** **1**. These results are different from the traditional Schottky junction, in which *J*_0_ and *V*_oc_ should slightly increase with *N*_D_, leading to a weakly decreased *V*_oc_ if the short‐circuit current density (*J*_sc_) remains constant. However, in this experiment, the *N*_D_ shows a strong effect on the *J*_0_ and *V*_oc_. In this viewpoint, the transport mechanism is dominated by the minority carrier diffusion driven by a similar p^+^n junction. Obeying the p^+^n junction model, the key factors that affect the device performances include the properties of the PEDOT:PSS and the interface management, i.e., conductivity, film‐formation and WF of PEDOT:PSS, the light‐trapping ability of the device, and the contact state of the PEDOT:PSS/Si interface. These will be discussed in detail in the following sections.

**Table 1:** Summary of *V*_oc_ and *J*_0_ extracted from the illuminated and dark *J–V* curves as well as the calculated values assuming a Schottky junction and an abrupt p^+^n junction for different *N*_D_. Reproduced with permission.^4^ Copyright 2015, Nature Publishing Group

*N*_D_ [cm^−3^]	*J–V*‐curves		Schottky junction		p^+^n junction	
	*J*_01_ [A cm^−2^]	*V*_oc_ [V]	*J*_0_ [A cm^−2^]	*V*_oc_ [V]	*J*_0_ [A cm^−2^]	*V*_oc_ [V]
4.9 × 10^14^	1.3 × 10^−11^	0.542	9.7 × 10^−10^	0.441	8.0 × 10^−12^	0.563
1.5 × 10^15^	3.1 × 10^−12^	0.564	6.8 × 10^−10^	0.450	3.4 × 10^−12^	0.585
1.4 × 10^16^	4.5 × 10^−13^	0.608	3.1 × 10^−10^	0.470	4.4 × 10^−13^	0.637
1.6 × 10^17^	3.2 × 10^−13^	0.634	4.6 × 10^−10^	0.458	4.1 × 10^−14^	0.696

### 2.2 PEDOT:PSS Modification

#### 2.2.1 Conductivity

PEDOT:PSS is widely used as the hole‐conducting polymer in organic devices, in which the conductive elements of PEDOT were well dispersed in the water soluble insulating polymer matrix of PSS. The conductivity of the pristine PEDOT:PSS solution is smaller than 1 S cm^−1^, but it can be improved to ≈1000 S cm^−1^ by adding cosolvents, dimethyl sulfoxide (DMSO), or ethylene glycol (EG). Moreover, secondary treatment could further improve the conductivity of PEDOT:PSS to several thousand S cm^−1^ by separating the conductive PEDOT and insulating PSS. It is believed that the improvement in conductivity of PEDOT:PSS is vital to the performance of PEDOT:PSS/n‐Si HSCs, enabling a higher fill factor (*FF*) and *J*_sc_.^30^, ^31^, ^32^, ^33^

**Figure 2:**
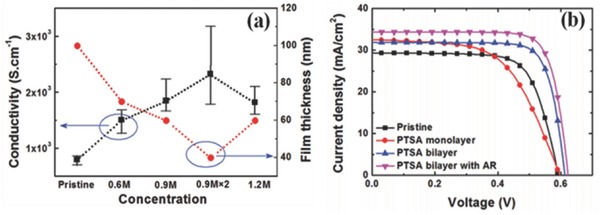
a) Conductivity and film thickness of PEDOT:PSS treated with various concentrations of PTSA and the number of the treatment cycle; b) *J–V* curves of PEDOT:PSS/n‐Si HSCs without and with PTSA/DMSO and PTSA/DMSO with ARC. Reproduced with permission.^33^

However, the underlying improvement in efficiency is rarely reported because most of the solution‐based treatments would deteriorate the interface quality of the PEDOT:PSS/n‐Si, making the loss outweigh the gain. It is worth mentioning that a gentle post‐treatment with p‐toluenesulfonic acid (PTSA) was developed by Liu et al. in which the conductivity of the PEDOT:PSS film was greatly improved while the interface of PEDOT:PSS/n‐Si could be well maintained.^33^ As shown in **Figure** **2**a,b, the incremental gain in conductivity of the PEDOT:PSS film was directly shown by the remarkable improvement in the *FF* and *V*_oc_.

#### 2.2.2 Film Forming and Coating Properties of PEDOT:PSS

**Figure 3:**
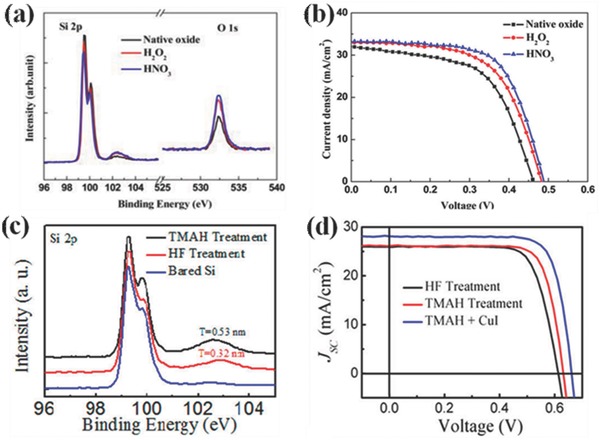
a) XPS spectra of the c‐Si surfaces and b) *J–V* curves of the PEDOT:PSS/Si HSCs treated with different oxidizing agents; c) XPS spectra in the Si 2p region collected from bare Si, HF treatment and TMAH treatment; d) *J–V* curves of the PEDOT:PSS/Si HSCs with HF treatment, TMAH treatment, and TMAH treatment & a capping layer of high work function CuI. Reproduced with permission.^35^ Copyright 2010, American Chemical Society.

For organic stacked HSCs, the performances are also critically influenced by the interfacial nature. A thin oxide layer cannot be avoided at the PEDOT:PSS/Si interface during the fabrication process.^34^ This thin oxide layer not only improves the wettability of the Si substrate, resulting in better coating of the PEDOT:PSS film, but also acts as a passivation layer to the Si surface and thus reduces interfacial recombination. As presented in **Figure** **3**, previous research showed that with proper treatment of the H‐terminated Si sample in a nitric acid (HNO_3_) or tetramethylammonium hydroxide (TMAH) solution, the thickness and valence states of the oxide layer can be well controlled in a good condition for achieving both better passivation and carrier tunneling.^35^

**Figure 4:**
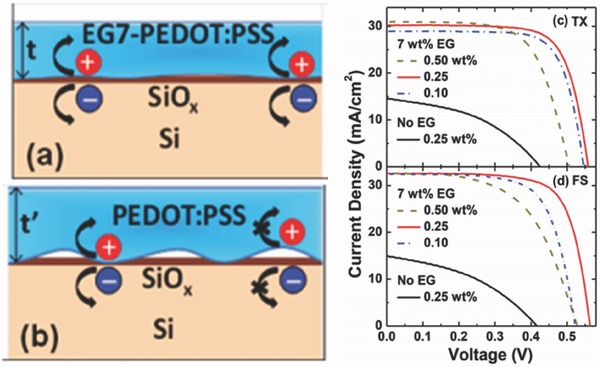
Schematic models of the PEDOT:PSS layer formation on SiO*_x_*/Si for a) EG7‐PEDOT:PSS and b) pristine PEDOT:PSS, added with 0.25 wt% surfactant (FS or TX); *J–V* curves of PEDOT:PSS/planar‐Si HSCs with 7 wt% EG and 0.10, 0.25, and 0.50 wt% c) TX and d) FS. Reproduced with permission.^42^

In addition, proper surfactants, such as Triton‐X100 (TX), fluorosurfactant (FS), etc. were usually added into the PEDOT:PSS solution prior to the spin‐coating process in order to improve its wettability.^36^, ^37^, ^38^, ^39^, ^40^, ^41^ With the aid of three‐dimensional chemical images, Leung and co‐workers observed that the sample without the addition of EG possesses a large number of microsized voids in the PEDOT:PSS/Si interface in comparison with a sample with 7 wt% EG, and the voids will severely affect the interfacial passivating quality and carrier collection capability.^42^ Through the addition of the optimal dose of surfactant (0.25 wt% FS), a minimal‐defect interface can be achieved with maximum contact area between the co‐solvent‐added PEDOT:PSS polymer and the Si substrate, leading to significant improvements in the PV performance, as shown in **Figure** **4**.

#### 2.2.3 WF of PEDOT:PSS

As mentioned in the transport mechanism section, the device performances are highly depended on the WF of PEDOT:PSS.^4^ As is reported, there are two normal ways to increase the WF of PEDOT:PSS films, i.e., modify the PEDOT:PSS solution by adding foreign materials,^43^, ^44^, ^45^ and coating high‐WF materials upon the PEDOT:PSS film.^35^, ^46^, ^47^ Sun and co‐workers first demonstrated that the WF of PEDOT:PSS can be tuned by adding an aqueous solution of perfluorinated ionomer (PFI).^44^ Due to the lower ionization potential level and higher dipole moment of PFI, a strong dipole between PEDOT:PSS and PFI was formed. With a 4% PFI addition, the WF of PEDOT:PSS was increased by ≈0.17 eV, resulting in an improvement in the efficiency from 8.2% to 9.9%. Similarly, it was reported that adding two‐dimensional cobalt sulfide (CoS) nanosheets into the PEDOT:PSS film can also enhance its WF.^45^

**Figure 5:**
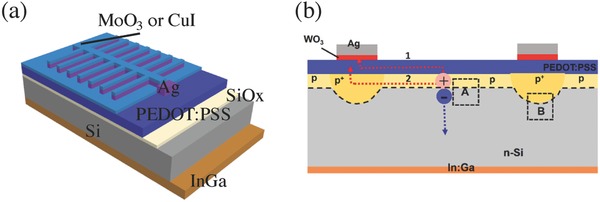
a) Device configuration of the PEDOT:PSS/Si HSC with a CuI or MoO_3_ capping layer; b) schematic diagram of photogenerated carrier separation and transportation mechanism with a WO_3_ interlayer between the Ag electrode and the PEDOT:PSS layer. Reproduced with permission.^47^ Copyright 2015, Elsevier.

Noting that adding foreign materials in the PEDOT:PSS solution may deteriorate its electrical property, coating high‐WF materials upon the PEDOT:PSS film without sacrificing its conductivity is thus a more efficient way to increase the WF of the PEDOT:PSS film. As shown in **Figure** **5**a, in 2014, Sun and co‐workers proposed that the capped MoO_3_ ARC layer can alter the effective WF of PEDOT:PSS films and then enhance the inversion effect in the Si underneath, leading to a stronger built‐in electric field. With this simple post‐processing method, they achieved an enhanced *V*_oc_ and efficiency of 630 mV and 13.8%, respectively, on planar PEDOT:PSS/n‐Si HSCs.^47^ Then, Yu and co‐workers introduced a high‐WF WO_3_ thin layer locally between the front Ag‐grid electrode and the PEDOT:PSS film in the PEDOT:PSS/n‐Si HSCs to form a dopant‐free selective emitter structure, as illustrated in Figure 5b.^48^ With the addition of this WO_3_ thin layer, the *V*_oc_ and *FF* of this kind of HSC was significantly improved due to the suppressed interfacial recombination and reduced contact resistance between the Ag electrode and PEDOT:PSS. He et al. used the more stable capping layer of copper iodide (CuI) as a high‐WF inducer to reinforce the inversion effect.^35^ Ultraviolet photoelectron spectroscopy (UPS) measurements indicated that the WF of PEDOT:PSS near the Si surface increased by 0.1 eV, which significantly improved the interface electrical passivation and strengthened the inversion layer. A competitive *V*_oc_ of 656 mV and a high *FF* of 78.1% were finally achieved, leading to a superior efficiency of over 14.3% for the planar PEDOT:PSS/n‐Si HSCs.

### 2.3 PEDOT:PSS/n‐Si CSCs on Structured Si Substrates

For planar PEDOT:PSS/n‐Si HSCs, although we obtain a competitive performance in *V*_oc_ and *FF* via the proper interfacial treatment or surface modification, the ultimate efficiency is still unsatisfied in comparison with the traditional c‐Si SC due to limited light‐trapping. The overall reflectivity in planar PEDOT:PSS/n‐Si HSC is still higher than 25% even though the organic layer could act as an ARC, resulting in a theoretical *J*_sc_ below 30 mA cm^−2^.^26^ Thus, effective light management, either ARCs on PEDOT:PSS layer or nanostructures on the Si surface, are essential for this kind of HSCs. Under the limitations of the mismatched reflection index of PEDOT:PSS (≈1.6) as well as the small room for adjustment in the thickness, which was restricted by the requirements of charge transporting and interfacial passivation, the effectiveness of the ARC design is not significant enough to constitutionally boost the optical absorption of the Si substrate. In comparison, nanostructured Si can provide not only excellent light‐trapping but also increased junction area due to its large specific surface area.

#### 2.3.1 Advanced Light‐Trapping Designs for Improved *J*_sc_

For the advanced Si nanostructures, such as nanopyramids, nanocones (NC), and nanocone‐nanopillar (NC‐NP) arrays, optimized light‐harvesting and broadband light absorption can be achieved through precise control of the shape and geometry.^49^, ^50^, ^51^, ^52^, ^53^, ^54^, ^55^, ^56^, ^57^, ^58^ A unique integrated NC‐NP dual‐structured array has been successfully realized by combining colloidal lithography with a multiple metal‐assisted chemical etching (MaCE) process.^52^ It was found that, for the same height, the NC‐NP dual‐structure provides efficient geometric confinement for the incident incoming photons and shows superior light trapping over the entire useful solar spectrum (375–1100 nm). As a result, a 20 µm‐thick c‐Si based hybrid SC textured by an NC–NP array can thus achieve a high *J*_sc_ of 32.8 mA cm^−2^ in comparison with the value of 23.8 mA cm^−2^ measured from the reference sample (300 µm thick flat c‐Si). Employing NW/micropyramid hierarchical structures, He and co‐workers further promoted the efficiency of PEDOT:PSS/n‐Si HSCs to beyond 11% with a *J*_sc_ as high as 34.5 mA cm^−2^.^55^

#### 2.3.2 Achievement of Full Contacts at PEDOT:PSS/structured‐Si

Nonetheless, most studies show that the PV performances of nanostructured SCs are greatly deteriorated by the high carrier recombination rate caused by the high density of defects at the surface or interface, which are associated with either the large surface area and/or the fabrication itself. This is not exception for PEDOT:PSS/n‐Si HSCs. Moreover, the implementation of the nanostructures in PEDOT:PSS/n‐Si hybrid cells is more challenging because the uniform PEDOT:PSS layer can rarely be conformably coated on the textured substrate due to its polymeric characteristics, and therefore, this leads to the poor junction quality of the polymer/silicon contact. This explains why the *V*_oc_ of the PEDOT:PSS/n‐Si hybrid cells based on nanostructured Si is always lower than that of the planar cells. Previous attempts to integrate the textured Si into hybrid SCs aimed at modifying the morphology of the surface textures to include a large open area and shallow depth for facilitating the deposition of PEDOT:PSS with enhanced coverage. Another strategy involved inserting a layer of small molecules between the textured Si and PEDOT:PSS to partially passivate the uncovered Si portions, suppressing charge recombination to some content.^59^, ^60^, ^61^, ^62^, ^63^, ^64^

**Figure 6:**
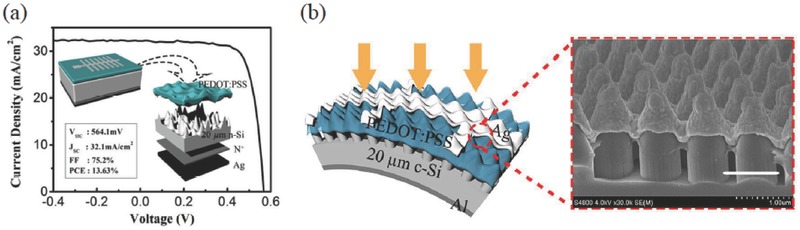
a) *J–V* curves of the 20 µm PEDOT:PSS/c‐Si HSCs with a MACE‐reconstructed Si NW structure. The inset shows the structure configuration; Reproduced with permission.^66^ Copyright 2015, American Chemical Society. b) structure configuration of 20 µm PEDOT:PSS/n‐Si HSCs with the NC–NP dual‐structure as a light‐trapping scheme. Reproduced with permission.^52^

Sun and co‐workers immersed high‐density Si NWs samples in phosphorus pentachloride solution to decrease the density of the NWs, showing that both a lower density of interfacial defects and a decreased majority carrier charge transfer velocity can be achieved. This treatment leads to the improved electrical output characteristics of HSCs.^50^ Similarly, Yu et al. used a thermal oxidation process to reconstruct the NW array to possess a low filling ratio, showing enhanced *V*_oc_ and *FF* for the corresponding devices. The low‐filling‐ratio Si‐NW/PEDOT:PSS heterojunction shows an enhanced built‐in potential and a strong inversion layer near the Si surface, which effectively suppresses the carrier recombination and reduces the leakage current of the SCs.^65^ Cai and co‐workers optimized the structure of the bottom pyramids via an acid isotropic etching method to ensure the pyramid‐structured Si surface can be fully covered by the PEDOT:PSS film, resulting in a *PCE* boost from 7.47% to 10.39%.^62^ He et al. reconstructed a conventional NW structure as hierarchical bowl‐like nanopores on a 20 µm c‐Si substrate, as shown in **Figure** **6**a.^66^ With omnidirectional light‐trapping performance over the entire solar spectrum, this structured thin‐film HSC (with a back‐side field design) shows a *J*_sc_ value comparable to that of its bulk counterpart and achieves an efficiency ≈13.6% due to the superior optical property and the improved heterojunction quality. A modified MACE reconstruction method was applied to produce an NC–NP dual‐structure on a thin‐film Si substrate, as shown in Figure 6b.^52^ The optimized NC–NP array can enhance the optical absorption of a 10 µm c‐Si close to the Lambertian limit, demonstrating huge potential in ultrathin SCs applications. In addition, the reconstructed NC structure can enable the PEDOT:PSS film to be well coated on the nanostructured Si with reduced interfacial recombination.

**Figure 7:**
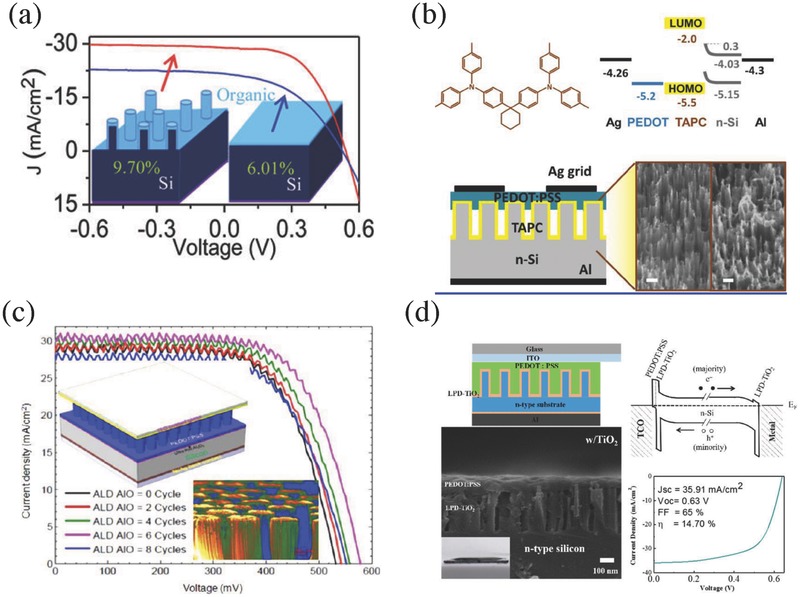
a) *J–V* characteristic curves of the core–shell hybrid SCs; Reproduced with permission.^67^ Copyright 2011, American Chemical Society. b) the molecular structures of TAPC, the device structure and cross‐sectional SEM image of Si‐NW/TAPC hybrid SC; ‘Reproduced with permission.^68^ Copyright 2013, American Chemical Society. c) *J–V* characteristic curves of the hybrid SCs with ALD‐processed aluminum oxide as passivation layer; Reproduced with permission.^70^ Copyright 2013, American Chemical Society. d) the structure configuration of PEDOT:PSS/structured‐Si hybrid SC with liquid‐phase processed TiO_2_ as an interface passivation layer, the energy band diagram, the SEM image of the Si/PEDOT:PSS interface, and the *J–V* curve. Reproduced with permission.^41^ Copyright 2016, American Chemical Society.

For interfacial passivation using an organic layer, Sun and co‐workers fabricated PEDOT:PSS/Spiro‐OMeTAD/Si‐NW HSCs with methyl groups terminating the dangling bonds of the NWs and spin‐coated Spiro‐OMeTAD as a buffer layer to form a core‐shell structure, which delivered an efficiency approaching 10%, as shown in **Figure** **7**a.^67^ Similarly, Yu et al. introduced an intermediate 1,1‐bis[(di‐4‐tolylamino)phenyl]cyclohexane (TAPC) small‐molecule layer into the PEDOT:PSS/Si‐NW interface to passivate the interfacial recombination centers and achieved an efficiency of over 13%, as shown in Figure 7b.^68^ With the additional TAPC interlayer, the PEDOT:PSS layer could be well incorporated into the NW surface to decrease the interfacial recombination. Meanwhile, the TAPC interlayer could effectively suppress the oxidation reaction between PEDOT:PSS and Si, which would improve the device performance and reliability. Meanwhile, the *V*_oc_ remained below 550 mV, which restricted further improvements in the efficiency. Most recently, Sun and co‐workers added an organic silane coupling agent, namely, 3‐glycidoxypropyltrimethoxydsilane (GOPS), into the PEDOT:PSS solution to improve the adhesion force between the organics and Si.^69^ The GOPS additive can chemically graft onto the nanostructured Si surface and thus suppress the surface charge recombination velocity at the nanostructured Si/PEDOT:PSS interface. With this easy method, a *V*_oc_ of 640 mV associated with a *PCE* of 14.1% was obtained.

Some researchers also showed promising results on inorganic dielectric layers for interlayer passivation, although the performance of the related devices was still insufficient due to the poor hole‐transporting property in these dielectric layers. Pudasaini et al. tried to use an ultrathin aluminum oxide layer to passivate the ordered Si NPs, showing effective interfacial passivation and enhanced *V*_oc_ on the SCs, as shown in Figure 7c.^70^ Rusli and co‐workers used low‐temperature ozone treatment to form an oxide layer on the nanostructured Si surface and then followed with short‐time HF treatment to partially remove this oxide, demonstrating reduced surface recombination for the final nanostructured‐Si/PEDOT:PSS system.^71^ Pei and co‐workers applied a liquid‐phase processed TiO_2_ as interlayer between nanostructured Si and PEDOT:PSS. The device structure and energy band alignment are shown in Figure 7d.^41^ With a hydrophilic TiO_2_/Si‐nanohole surface, the PEDOT:PSS solution could easily flow into the spacing of the close‐packed nanoholes. Combined with the passivation effect of the TiO_2_ layer, the HSCs with nanohole‐textured Si achieved an efficiency and *V*_oc_ of 14.7% and 0.63 V, respectively. Although the TiO_2_ has a mismatched valence band, the photogenerated holes could be extracted effectively by the PEDOT:PSS layer through the trapping states in the TiO_2_ film.

**Figure 8:**
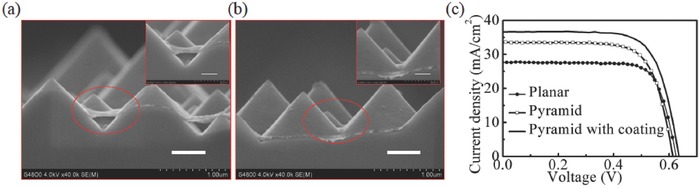
a) Cross‐sectional scanning electron images of the as‐fabricated PEDOT:PSS/n‐Si junction on textured‐Si substrate without DEP coating and b) with DEP coating (the DEP layer was removed with an acetone solution to capture clear images). Scale bars, 500 nm in (a) and (b) and 250 nm for the insets, respectively. c) Light *J–V* characterization of the HSCs with a planar n‐Si substrate and the pyramidal n‐Si substrate with and without a DEP coating. Reproduced with permission.^24^

Finally, a fully conformal and close contact between the PEDOT:PSS thin‐film and pyramid‐textured Si was eventually achieved by post‐coating with a water‐insoluble phthalic acid ester, diethyl phthalate (DEP), onto the PEDOT:PSS layer in an as‐fabricated device. By applying an external acting force on the suspended polymeric PEDOT:PSS, in situ changes from half‐contact to full‐contact between the PEDOT:PSS and Si pyramids (even at the valley regions) was clearly observed by scanning electron microscopy (SEM) measurements, as shown in **Figure** **8**.^24^ This change in morphology results in a greatly decreased surface recombination velocity from 300 to 100 cm s^−1^ at the PEDOT:PSS/Si‐pyramids interface, leading to a significantly high external quantum efficiency (*EQE*) approaching 90% in wavelengths ranging from 400 to 1000 nm. The *PCE* was directly prompted from 14% to 16.2% with a typical *V*_oc_ exceeding 630 mV. In addition, the relationship of the rear‐ and front‐surface recombination velocities on the *V*_oc_ of PEDOT:PSS/Si hybrid HSCs were thoroughly investigated experimentally and theoretically. Their findings clearly clarify the reasons that PEDOT:PSS/textured‐Si hybrid SCs show inferior *V*_oc_ and efficiency over planar counterparts in previous reports, and further suggest a pathway to fully explore the efficiency potential of the PEDOT:PSS/n‐Si hybrid SCs.

In brief, we can draw a conclusion that proper nanostructures with decreased surface area, smooth surfaces, impedance‐matching geometry and enhanced light‐trapping properties are essential for both large *J*_sc_ and *V*_oc_, and the inefficiency in the carrier collection cannot be overcome unless a fully conformal and close contact between the PEDOT:PSS and textured Si‐surface is achieved. Therefore, new strategies and insights are needed to improve the junction quality and performance of PEDOT:PSS/textured‐Si HSCs.

### 2.4 Efficiency Evolvement and Prediction of PEDOT:PSS/Si CSCs

**Figure 9:**
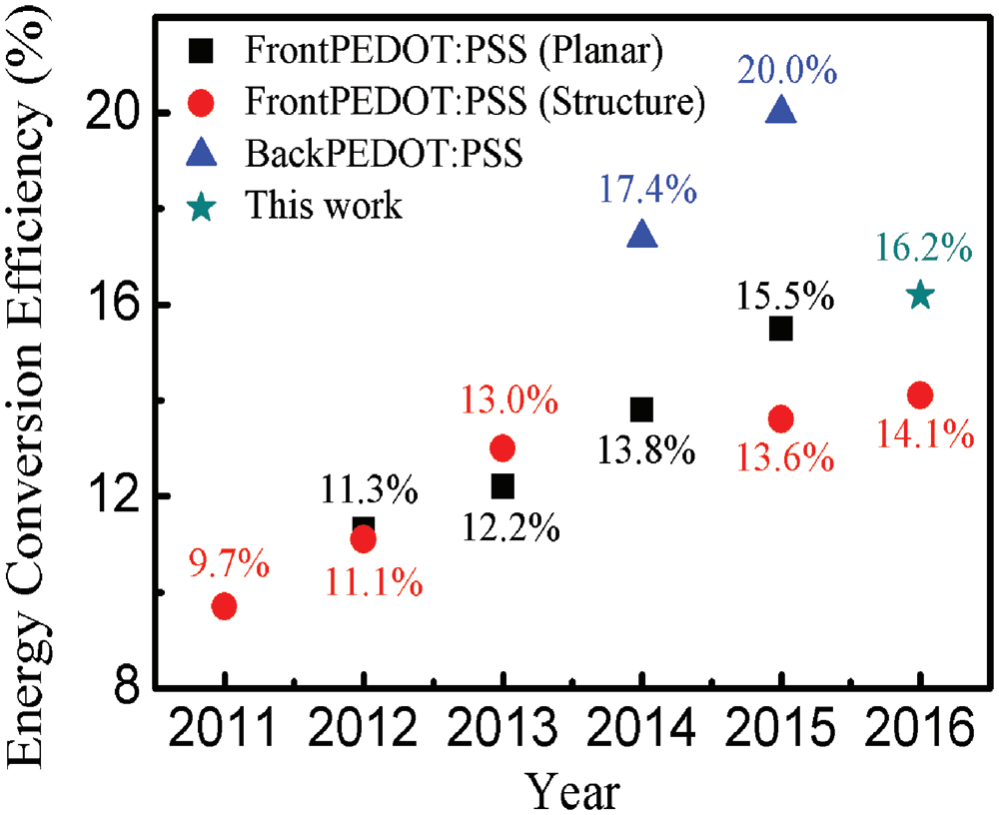
Efficiency evolution of PEDOT:PSS/n‐Si HSCs over time. FrontPEDOT:PSS means the PEDOT:PSS is placed upon the top‐surface of the SC where the active light shines, whereas the PEDOT:PSS in the BackPEDOT:PSS structure is only used as a back‐surface field (the main junction in front is still a high‐temperature diffused Si‐emitter). Reproduced with permission.^24^

The evolution of the efficiency of the PEDOT:PSS/n‐Si HSCs is shown in **Figure** **9**,^24^, ^47^, ^56^, ^66^, ^67^, ^68^, ^69^, ^72^, ^73^, ^74^, ^75^ in which some remarkable progress is addressed. Here, we only consider the conventional front PEDOT:PSS HSCs because the PEDOT:PSS in this kind of structure serves as a main junction and can be entirely attributed to the dopant‐free concept. We can see that the experimental *PCE* is 16.2%, which is still far below to traditional Si‐based homojunction SCs. Therefore, it is practical to thoroughly analyze all the opto‐electrical loss mechanisms to determine the margin for future improvement. First, the parasitic absorption in PEDOT:PSS that degrades the effective optical generation in the Si layer cannot be avoided in PEDOT:PSS/Si HSCs. The absorption ability of the PEDOT:PSS layer depends not only on the thickness of the PEDOT:PSS layer but also on the amount of PEDOT because PSS is almost colorless. The conductivity of PEDOT:PSS can be attributed to the PEDOT. Thus, it is meaningful to consider the parasitic absorption in the PEDOT:PSS, the light‐trapping ability of the device (thickness of PEDOT:PSS) and the conductivity of PEDOT:PSS, aiming to achieve a high‐performance device. In addition to the parasitic absorption in PEDOT:PSS, the efficiency of this kind of HSCs is also restricted by many other factors.

**Figure 10:**
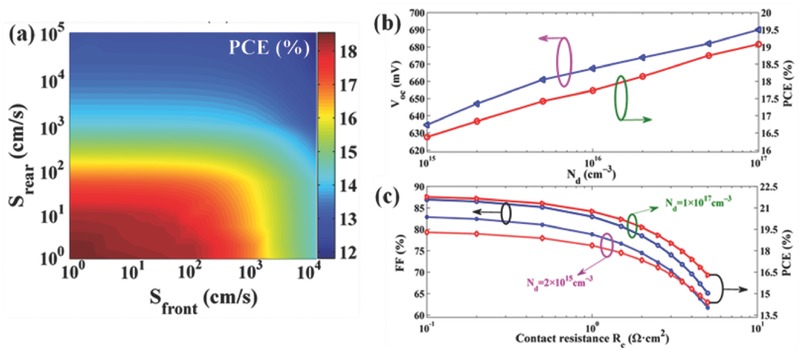
a) Simulated *PCE* versus front/rear recombination velocities of the n‐Si interface (*S*_front_/*S*_rear_) for the PEDOT:PSS/n‐Si HSCs under *N*_d_ = 2 × 10^15^ cm^−3^; b) *V*_oc_ (blue line) and *PCE* (red line) as a function of *N*_d_; c) *FF* and *PCE* versus *R*_c_ for the PEDOT:PSS/n‐Si HSCs. Reproduced with permission.^60^ Copyright 2017, American Chemical Society.

Based on this, Yang et al. predicted the efficiency of PEDOT:PSS/n‐Si HSCs under different front‐sided recombination velocities, doping concentrations (*N*_d_) and contact resistances (*R*_c_), as shown in **Figure** **10**.^60^ As shown in Figure 10a, if the rear interface is poorly passivated (*S*_rear_ > 1000 cm s^−1^), the front interface has less effect on the efficiency. For the Si substrate with *N*_d_ = 2 × 10^15^ cm^−3^, the efficiency is expected to exceed 18% as soon as the double‐sided recombination velocities are kept below 10 cm s^−1^. Meanwhile, with the *N*_d_ increasing to 1 × 10^17^ cm^−3^, the predicted *V*_oc_ and efficiency beyond 690 mV and 19%, respectively, is possible even for a medium quality of passivation (i.e., *S*_front_ = 100 cm s^−1^ and *S*_rear_ = 50 cm s^−1^), as shown in Figure 10b. Moreover, as shown in Figure 10c, the efficiency can be further increased to 21.30% when the contact resistance decreases to 0.5 Ω·cm^2^ under *N*_d_ = 1 × 10^17^ cm^−3^ based on high‐quality passivation.

In a PEDOT:PSS/n‐Si HSC, the *V*_oc_ is determined by the quasi‐Fermi level splitting of electrons and holes in the whole device under illumination, which is affected not only by the band alignments at the front and rear contacts but also by the electronic states related to the unpassivated interface states. Interface engineering of the PEDOT:PSS/n‐Si junction is still the highest priority toward improving the cell performance, because it is rationally interpreted as a driving force, and we believe it is far from being fully optimized. However, the achievement of concurrent improvements in the passivation and carrier collection at this interface is not easy, wherein various impacts including the reduction of interfacial trapping states, the minimization of the contact resistivity, and the precise tuning of the WF of PEDOT:PSS and other materials must be considered simultaneously. New strategies using highly controllable interface chemical treatments and stable functional materials with the capability to approach the abovementioned multiple targets are eagerly needed to improve the junction quality and performance of PEDOT:PSS/n‐Si HSCs.

### 2.5 Stability Improvement

In addition to efficiency, the operational stability, which is determined by the time it takes before the performance of a device degrades to an unacceptable level, also needs to be considered. So far, efficiency degradation is a common problem in organic and some related high‐WF TMO‐containing HSCs. In commercially available c‐Si‐based solar modules, performance degradation is traced back to the degradation of the glass, contacts, and cabling, whereas the Si substrate shows long‐term stability. However, for the organic SCs, structural and chemical variations may occur in both the organic PEDOT:PSS and the underlying thin tunneling oxide layer, especially under operational conditions involving oxygen, water and ultraviolet (UV)‐light.

**Figure 11:**
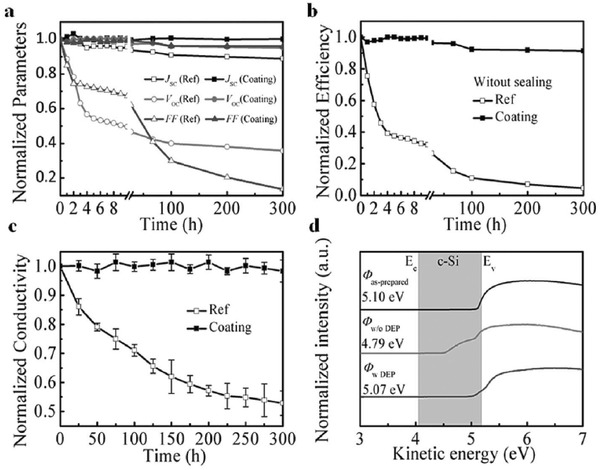
a) Normalized photovoltaic parameters and b) efficiency degradation of the HSCs with and without the DEP coating; c) stability in the conductivity of the PEDOT:PSS films with and without the DEP coating. ‘Ref' represents cells without the DEP coating and ‘Coating' represents cells with the DEP protection; d) UPS was used to measure the WF of the as‐prepared PEDOT:PSS and the stored PEDOT:PSS with and without DEP coating. The shaded area in (d) represents the bandgap of n‐Si. Reproduced with permission.^24^

Comparison of the PEDOT:PSS/n‐Si HSCs with and without an antimoisture coating for long‐term storage is shown in **Figure** **11**.^24^ For the unprotected cell, quick degradation of the *V*_oc_, *FF* and efficiency is observed (depicted in hollow masks in Figure 11a,b when this SC was kept in atmosphere for over 4 h. There are two main moisture‐related degradation routes that appeared in this HSC. One is related to the interfacial tunneling oxide layer. For the PEDOT:PSS/n‐Si interface, a thin oxide layer seems inevitable during the fabrication process. Jäckle et al. found that by increasing the interfacial oxide thickness, the *J–V* curves of the PEDOT:PSS/n‐Si HSCs exhibit a so‐called s‐shape with a very low efficiency.^76^ The growth of this oxide layer would be exacerbated upon exposure to moisture and high temperatures, and the charge carrier separation and transport would thus be greatly affected. The second degradation route of the PEDOT:PSS/n‐Si HSCs in atmosphere is the degradation of the PEDOT:PSS film itself. In contrast to other organic conducting polymers that are sensitive to both temperature and humidity, the PEDOT:PSS film is considered to be temperature stable. Thus, the main problem affecting its stability is humidity, arising from the hydroscopic behavior of the PSS group. As presented by He et al. in Figure 11c,d,^24^ the conductivity and the WF of the PEDOT:PSS film rapidly degraded when the film was exposed in the atmosphere. However, for the PEDOT:PSS layer coated with an antimoisture film, negligible degradation appears. The antimoisture film‐coated PEDOT:PSS/n‐Si HSC also shows negligible degradation in the device performances even with over 300 hours of storage (depicted in the solid mask in Figure 11a,b).

**Figure 12:**
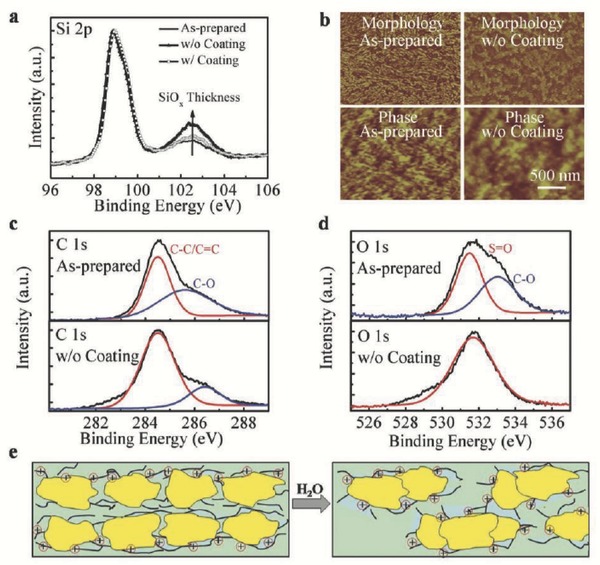
a) XPS spectra in the Si 2p region for the interfacial Si oxide tunneling layers of the as‐prepared PEDOT:PSS/n‐Si and long‐term stored samples with and without the DEP coating; b) AFM morphology and phase images of the fresh as‐prepared PEDOT:PSS film and the film stored over a long term without the DEP coating. Scale bars, 500 nm; c) C 1s and d) O 1s XPS spectra of the as‐prepared PEDOT:PSS film and the film without the DEP coating stored over a long term; e) schematic illustration of the PEDOT:PSS film on the performance degradation after long‐term storage. The yellow parts represent the PEDOT and the long chain of the PSS. Reproduced with permission.^24^

The degradation of the electrical performance of the PEDOT:PSS films likely relates to the structure and morphology changes of the PEDOT and PSS chains. Detailed morphology and X‐ray photoelectron spectroscopy (XPS) characterization of the PEDOT:PSS film as‐prepared and with long‐term exposure in air were investigated by He et al. and are shown in **Figure** **12**.^24^ Atomic force microscopy (AFM) phase imaging shows that with long‐term exposure, the PEDOT:PSS film exhibits almost disconnected PEDOT chains with increased PSS cladding. Larger inter‐grain distances and thicker PSS barriers can discourage charge hopping between the highly conductive PEDOT‐rich grains, leading to a lower conductivity. XPS characterization shows that the disintegration of the same C—O bonding appears in aged PEDOT:PSS films; thus, the lone pairs of oxygen atoms cannot participate in the conjugation of PEDOT resulting in a decrease in the conductivity. The growth of the oxide layer combined with the increased resistance in the PEDOT:PSS film leads to the increase in the series resistance (*R*_s_) of the HSCs and thus decreases *FF*.

Two kinds of mainstream methods can be used to prevent the degradation of HSCs.^76^, ^77^, ^78^, ^79^ One is to encapsulate the PEDOT:PSS film with an antimoisture capping layer, such as Al_2_O_3_, TiO_2_, or a water‐insoluble ester. The second is to modify the PEDOT:PSS layer to reduce its water absorption performance. The detailed PEDOT:PSS modification evolution is presented in the previous discussion and is performed mainly by reducing the concentration of PSS in the PEDOT:PSS film using an effective acid treatment. For the encapsulation process, the principle of selecting the capping layer should focus on a high transparency and a low water vapor transition rate. With low‐temperature plasma‐enhanced atomic layer deposition (ALD), Schmidt et al. deposited a pinhole‐free, highly uniform and conformal Al_2_O_3_ film upon a PEDOT:PSS film, showing that the ALD encapsulation process can effectively prevent the performance degradation of this organic‐containing HSC without harming the PV performance.^79^ Similar results were also reported by Liu^33^ et al. and He^24^ et al. in which TiO_2_ and water‐insoluble phthalic acid ester were used as encapsulation layers.

## 3 TMO/Si HSCs

HSCs featuring TMOs/n‐Si contacts have received great attention due to their advantages of low opto‐electrical losses and a high efficiency potential. At present, 22.5% efficiency has been achieved for the HSCs with an MoO*_x_*/a‐Si:H(i)/n‐Si/a‐Si:H(i)/a‐Si:H(n) core structure.^18^ In this review, we focus particularly on three typical TMOs (namely, MoO*_x_*, VO*_x_*, and WO*_x_*), in which the material characteristics including WF, conductivity, oxygen vacancy defects, bandgap, etc., and their effects on the performances of TMOs/n‐Si HSCs are thoroughly investigated.^80^, ^81^, ^82^, ^83^, ^84^, ^85^, ^86^, ^87^, ^88^, ^89^ In addition, the interfacial passivation quality of TMOs/Si is also studied. Although the transport mechanism of this kind of HSC is not clear completely, we attempt to provide a possible explanation of the carrier transport and hope the results in this section can help guide the development of high efficiency TMOs/n‐Si HSCs.

### 3.1 Transport Mechanism of TMO/Si HSCs

**Figure 13:**
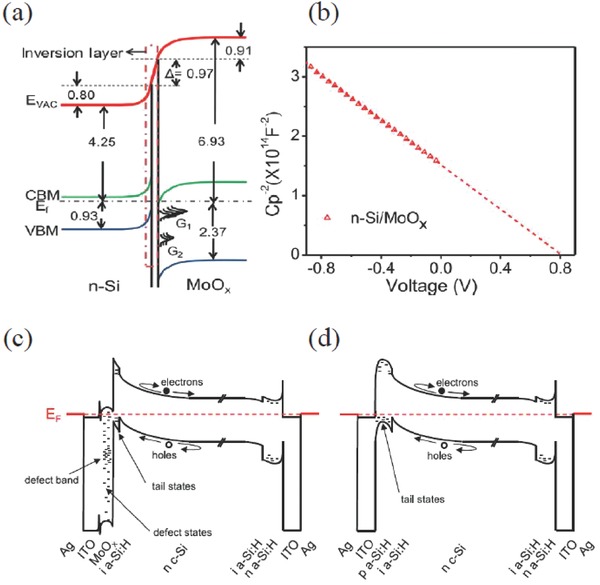
a) Energy‐level diagram for MoO*_x_* on an n‐Si substrate; b) *C–V* curve of a device with the Ag/MoO*_x_*/n‐Si/Al structure; Reproduced with permission.^90^ energy band diagrams for n‐Si HSC with a hole‐selective c) MoO*_x_* contact and d) a standard p‐type a‐Si:H emitter. Reproduced with permission.^91^ Copyright 2014, AIP Publishing LLC.

Although TMOs have already been experimentally demonstrated as potential alternatives to replace p‐doped a‐Si:H in traditional HJT SCs owing to their high WFs and hole extractive capabilities, the transport mechanisms of the TMOs/n‐Si heterocontacts have not been well explored. Sun et al. examined the energy level bending of MoO*_x_*/n‐Si heterocontacts by UPS and XPS measurements. In this model, they noted that a remarkable interface dipole (≈0.97 eV) is formed in association with band bending (≈0.80 eV) at both sides of the interface to maintain thermodynamic equilibrium, as shown in **Figure** **13**a,b.^90^ Gerling et al. proposed that TMOs tend to induce an inversion layer on the n‐Si surface due to their particularly large WFs (>5.5 eV), forming selective contact for hole extraction. The energy mismatch defined by the difference between the WF of the TMOs and the Fermi level of the n‐Si is the driving force for the Fermi level equilibration.^3^, ^84^ The process of energy‐band equilibration can be expressed as follows: 1) electron transfer from the n‐Si valence band (≈5.17 eV) into the deep oxygen vacancy (defect) states in the TMO is positive; 2) the valence band of n‐Si is close to the Fermi level, forming a p^+^ region after the electron was removed. In addition, the negative charges (excess oxygen) fixed in the TMO layers help to reinforce the inversion‐effect, which has been verified by the oxygen ratio. Since band bending cannot occur indefinitely, the n‐Si surface reaches a strong inversion condition up to a high built‐in voltage; 3) the large difference between the WF of the TMOs and the Fermi level of the n‐Si (≈1.2 eV) cannot fulfill the requirement of the Fermi level equilibrium, and thus, a negative dipole was assumed in the TMO/n‐Si interface.

As shown in Figure 13c,d, Javey and co‐workers compared the energy band diagram of the HSCs with the MoO*_x_* hole contact to p‐type a‐Si:H contact.^91^ The common feature of the double contacts is the formation of an inversion layer in the surface of the n‐Si substrate, accompanied by a large barrier for electrons resulting from band bending in the n‐Si and the conduction band offset between n‐Si and a‐Si:H. For both cases, n‐type MoO*_x_* and p‐type a‐Si:H both provide very similar situations for hole extraction, i.e., holes must first cross the barrier resulting from the valence band offset between n‐Si and a‐Si:H and then transit through the a‐Si:H tail states or oxygen vacancy‐derived defect states in MoO*_x_* into the degenerately doped ITO front electrode. In contrast, the barrier is controlled by the WF of the MoO*_x_* contact, while it is controlled by the doping concentration for the p‐type a‐Si:H contact.

### 3.2 M^+^‐Related WF and Conductivity of TMOs

The WF and conductivity of the TMOs are mainly determined by the number of oxygen deficiencies, which is influenced by the substrates, fabrication methods, post‐deposition treatments, etc.^92^, ^93^, ^94^, ^95^ The TMO can be fabricated via thermal evaporation,^18^ ALD,^87^ and solution processes.^88^ For the thermal evaporation method, the TMOs can be deposited from a tantalum boat at < 8 × 10^6^ mbar at a rate < 0.1 Å/s. The ALD method aims to overcome the nonconformal, porous ultrathin (<10 nm) films on textured surfaces. Taking MoO*_x_* for example, MoO*_x_* can be deposited by a plasma‐assisted ALD process using (NtBu)_2_(NMe_2_)_2_Mo as the Mo precursor and O_2_ plasma as the oxidant at low temperatures down to 50 °C. Meanwhile, the oxygen vacancy concentration of MoO*_x_* via solution processes is reduced by annealing the as‐deposited MoO*_x_* films in an ambient environment at temperatures exceeding 100 °C, which exhibits a contact resistivity and passivation quality on c‐Si wafers comparable to those found for the thermal evaporation method. In addition, with the introduction of a carefully controlled O_2_ partial pressure during the evaporation process, the WF of the TMOs can be increased, which reduces the O‐deficiency in‐gap state energy, making it more suitable for high‐performance HTL. Here, MoO*_x_* is used for illustration and discussion.

**Figure 14:**
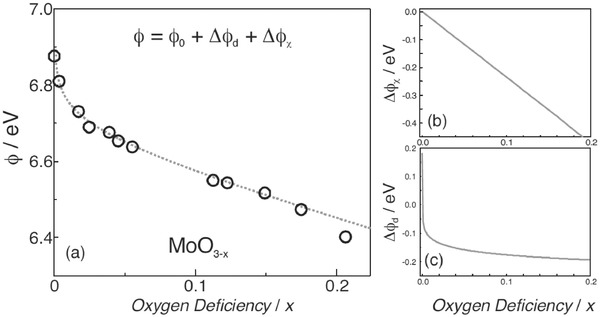
a) WF, b) electronegativity contribution, and c) donor state contribution of MoO*_x_* versus oxygen deficiency/*x*. The circles in (a) are the experimental data points, and the dashed curve is the predicted trend from Equation (2). Reproduced with permission.^92^

The WF versus the O deficiency, as determined from the UPS spectra, is shown in **Figure** **14**a.^92^ With increasing oxygen deficiencies, the WF of MoO*_x_* initially decreases rapidly and then follows a more gradual, nearly linear decreasing trend. This phenomenon can be explained by Equation (2)

(2) $$\varphi =\varphi_{0}+\Delta \varphi_{\chi }+\Delta \varphi_{d}$$

The expression for WF can explain this phenomenon, where ϕ is the measured WF, ϕ_0_ is the WF of the stoichiometric oxide, Δϕ_χ_ is the change in WF caused by an increase in the concentration of low electronegativity cations, and Δϕ_d_ is the change in WF caused by an increase in the density of donor states. The model stipulates that the WF decrease that accompanies the O‐deficiency is a result of the formation of lower electronegativity cations and the increase in the density of occupied states close to the Fermi level.

The conductivity of MoO*_x_* films has been shown to vary by more than ten orders of magnitude during the transition from the insulating MoO_3_, with reported conductivities as low as 10^−7^ S cm^−1^, to the semi‐metallic MoO_2_, which exhibits conductivities in the range of 10^4^ S cm^−1^. Gains in the conductivity are typically weighed against the transparency and WF, both of which are found to decrease with a decrease in the oxidation state.

**Figure 15:**
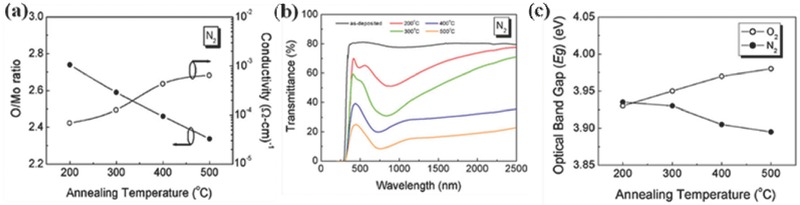
a) Annealing temperature dependence of the O/Mo ratio and conductivity of MoO*_x_* film in N_2_; b) transmittance spectra of MoO_3_ films annealed at various temperatures in N_2_; c) variation of the optical band gap of MoO_3_ films with annealing temperatures in N_2_ and O_2_. Reproduced with permission.^93^ Copyright 2008, Elsevier.

**Figure** **15** depicts the effects of the annealing temperature on the O/Mo ratio and the conductivity, transparency, and optical band gap (*E*_g_) of the MoO_3_ film in N_2_.^93^ A decline in the O/Mo ratio from 2.74 to 2.33 demonstrates that the films formed at high temperatures are more nonstoichiometric with lower optical transmission and *E*_g_. The conductivity of MoO*_x_* films increases as the annealing temperature increases from 200 to 500 °C. In contrast, annealing in O_2_ leads to the opposite results. Meanwhile, the substrates also influence the conductivity of the TMOs. Dark resistance measurements found sheet resistances of ≈5 × 10^12^ Ω sq^−1^ for MoO_3_/glass and ≈1 × 10^4^ Ω sq^−1^ for MoO_3_/n‐Si. The large difference between both measurements can be explained by the formation of an inversion layer on the Si surface, acting as a p‐type contact with the n‐Si substrates. The measured sheet resistance for MoO*_x_*, VO*_x_*, and WO*_x_* on glass and Si substrates are summarized in **Table** **2**.^96^

**Table 2:** Sheet resistance (*R*_sh_) of the oxides calculated from the transfer length method (TLM)

Oxide	Sheet resistance (*R*_sh_) [Ω sq^−1^]	
	Glass substrate	Silicon substrate
V_2_O*_x_*	3.3 × 10^9^	5.8 × 10^3^
MoO*_x_*	5 × 10^12^	1.0 × 10^4^
WO*_x_*	1.1 × 10^9^	1.2 × 10^5^

### 3.3 TMOs/n‐Si Interface

**Figure 16:**
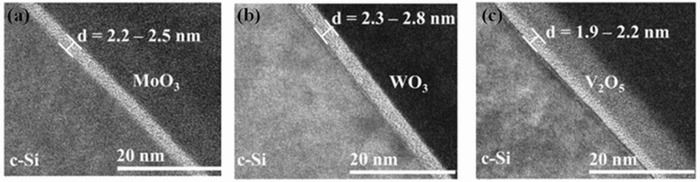
HR‐TEM images of the ITO/TMOs/n‐Si heterostructures showing an interlayer between n‐Si and a) MoO_3_, b) WO_3_, and c) V_2_O_5_. Reproduced with permission.^3^ Copyright 2016, Materials Research Society.

Analysis of the chemical states at the TMOs/n‐Si interface is vital to the surface passivation and transport mechanisms of TMOs/n‐Si heterojunctions. As shown in **Figure** **16**, detailed images of the as‐fabricated ITO/TMOs/n‐Si structures by HR‐TEM reveal the presence of an amorphous and relatively uniform silicon oxide layers. The maximum interlayer thickness is 2.8 nm (WO_3_), 2.5 nm (MoO_3_), and 2.2 nm (V_2_O_5_).^3^

**Figure 17:**
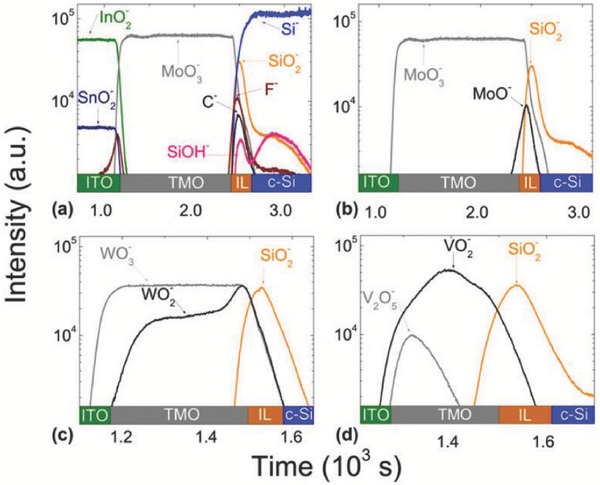
ToF‐SIMS depth profile for the ITO/TMOs/n‐Si heterostructures showing an SiO_2_^−^ signal at the interface between n‐Si and a,b) MoO_3_, c) WO_3_, and d) V_2_O_5_. For each TMO, the related reduced species (MoO^−^, WO_2_^−^, VO_2_^−^) are also detected near the interface. Reproduced with permission.^3^ Copyright 2016, Materials Research Society.

The composition of this oxide layer was further qualitatively determined as sub‐stoichiometric SiO*_x_*_≈1.5_ species (Si^3+^) by ToF‐SIMS and XPS analyses shown in **Figure** **17**.^3^ The formation of an SiO_2_^−^ layer is likely related to the chemical reaction with the TMOs during deposition; the feasibility of these reactions is given by their negative Gibbs formation energies. The silicon oxide peak is detected in the WO_3_/n‐Si, MoO_3_/n‐Si and V_2_O_5_/n‐Si interfaces in these figures at similar intensities and is associated with reduced TMOs species located adjacent to the interface (WO_2_, MoO, and V_2_O_4_). For vanadium oxide in particular, the reduction reaction extends over the thin V_2_O*_x_* layer, and the VO_2_^−^ species surpass V_2_O_5_^−^ in intensity. Reasonable passivation and good contact properties have been achieved and demonstrated with another kind of SiO*_x_* interlayer. The SiO*_x_* interlayer formed during standard RCA processing can now be used intentionally as a passivation interlayer. The *V*_oc_ and efficiency of the Ag/ITO/MoO*_x_*/SiO*_x_*/n‐Si/SiO*_x_*/Poly‐Si(n^+^)/Ag cell (2 × 2 cm) are at 637 mV and 16.7%, respectively.^97^ Further increasing the thickness of the SiO*_x_* interlayer demonstrated a negligible influence on the surface passivation as well as the current transport performance.^98^ Thus, many opportunities remain for tuning the properties of the SiO*_x_* interlayer.

**Figure 18:**
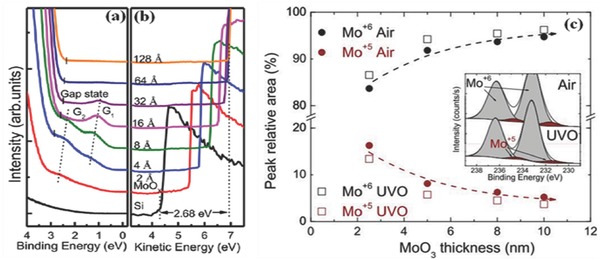
UPS spectra of the incremental MoO*_x_* deposition thickness on an Si substrate. a) A close‐up of the valence band region with respect to the Fermi level; b) secondary electron cutoff region, the vacuum level difference between pristine Si and the thick layer of MoO*_x_* is noted; Reproduced with permission.^90^ c) relative content of Mo^+6^ and Mo^+5^ oxidation states for different MoO_3_ film thicknesses and postdeposition treatments. Reproduced with permission.^96^ Copyright 2015, MDPI.

As shown in **Figure** **18**, in situ thickness‐dependent UPS and XPS measurements have been conducted to obtain an electronic structure at the interface between MoO*_x_* and n‐Si.^90^ The bottom spectra are photoemissions from the Si substrate, where the valence band maximum (VBM) and the vacuum level (VL) are located at 0.93 eV and 4.25 eV, respectively. Upon depositing MoO*_x_*, the spectral features of n‐Si are slightly attenuated, and the VL increases. Such a large VL shift points to electron transfer from n‐Si to MoO*_x_*. Finally, the shift becomes saturated for thicknesses larger than 64 Å, and the VL and VBM are located at 6.93 eV above and 2.37 eV below the Fermi level, respectively.

For a thin MoO*_x_* layer, additional gap states (G_1_ and G_2_) below the Fermi level can be observed, as marked in Figure 18. G_1_ (G_2_) is associated with singly (doubly) occupied Mo 4d orbitals. The gap state G_1_ appears at 4 Å, demonstrating a rather broad peak width. When the MoO*_x_* thickness increases to 64 Å, the G_1_ peaks decrease due to the interface state concealing the bulk‐like MoO*_x_* over‐layers. The peak attributed to gap state G_2_ is only visible for coverage from 2 to 8 Å, with a comparable low intensity and a shift toward a lower binding energy.

The elemental content of Mo^+6^ and Mo^+5^ cations at the TMOs/n‐Si interface is shown in Figure 18c.^96^ For the air‐exposed films, Mo^+6^ content increases with film thickness and stabilizes at a value of ≈94%, while Mo^+5^ content inversely decreases to ≈6%. This phenomenon and presence of the gap states have been thoroughly investigated for Si substrates and several metallic substrates, and they have finally been attributed to thermodynamically driven redox reactions at the MoOx/substrate interface. Similarly, UV‐ozone treated samples follow the same trend but with a larger Mo^+6^ content due to ozone oxidation.

### 3.4 Contact Resistivity and Passivating Quality of TMOs/n‐Si

The carrier‐selective properties of TMO‐based contacts on n‐Si surfaces are mainly evaluated via simultaneous consideration of the contact resistivity (ρ_c_) and the contact recombination parameter (*J*_0c_). Note that current flows across the metal/TMO and TMO/n‐Si interfaces, including the induced inversion layer on n‐Si, such that *ρ_c_* is the sum of the multiple contributions.

**Figure 19:**
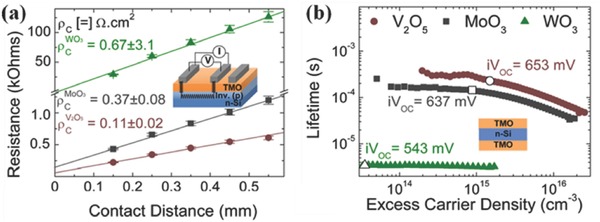
a) The *ρ_c_* values of Au/TMO/n‐Si systems extracted from the *C*–*V* responses in a TLM array; b) effective carrier lifetime of TMO/n‐Si/TMO configurations as a function of excess carrier density, where the implied‐*V*_oc_ values of three samples were also marked in the figure. The thicknesses of the three TMOs are 20 nm. Reproduced with permission.^3^ Copyright 2016, AIP Publishing LLC.

Bullock et al. demonstrated that the ρ_c_ and *J*_0c_ of MoO_3_ on n‐Si (2.1 Ω cm) are quite high, though still applicable to high efficient c‐Si SC designs, with optimum values of 30 mΩ cm^2^ and 300 fA cm^−2^, respectively.^99^ As shown in **Figure** **19**, Gerling et al. extracted ρ_c_ values of 110 mΩ cm^2^ (V_2_O_5_), 370 mΩ cm^2^ (MoO_3_), and 670 mΩ cm^2^ (WO_3_), all of which are close to the target series resistance values for most c‐Si SCs (0.1–0.5 Ω cm).^3^ The lifetime values at 1 sun illumination are 240, 142, and 4.5 µs for V_2_O_5_, MoO_3_, and WO_3_, respectively, which translate into implied‐*V*_oc_ values of 653 (V_2_O_5_), 637 (MoO_3_), and 543 mV (WO_3_), indicating a certain degree of surface passivation. Shen and co‐workers measured the *ρ_c_* values for moderately doped n‐Si (1–2 Ω cm) with WO_3_, MoO_3_, V_2_O_5_ and the results are 840, 86, and 34 mΩ cm^2^, respectively.^83^ Correspondingly, the implied‐*V*_oc_ increases from 575 (WO*_x_*) to 618 (MoO*_x_*) and 623 mV (V_2_O*_x_*), demonstrating an increased ϕ_0_ (the equilibrium dark band bending) and a reduced carrier recombination rate on the n‐Si surface. Without using both the intrinsic a‐Si layer and the TCO contact layer, Gerling and co‐workers explored V_2_O*_x_* capped with a thin Ni layer as a hole‐selective layer. With a 40 nm thick V_2_O*_x_* layer, they obtained a saturation current density of 175 fA·cm^−2^ and ρ_c_ below 115 mΩ cm^2^ based on figures of merit for Ni/V_2_O*_x_*/n‐Si samples.^98^

**Figure 20:**
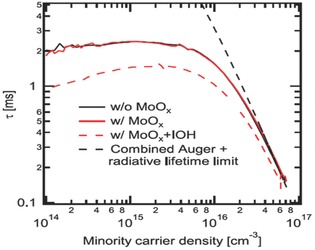
Minority carrier lifetime measurements before and after MoO*_x_* thermal evaporation and after IOH sputtering. Reproduced with permission.^91^ Copyright 2014, AIP Publishing LLC.

It is well known that the presence of an a‐Si:H layer at the interface may offer excellent surface passivation.^100^ The passivation stack of a‐Si:H/a‐Si:H (doped) double layers can always provide a lifetime ranging from 2–3 ms. **Figure** **20** shows the effective minority carrier lifetime of a‐Si:H‐passivated Si substrates before and after the evaporation of MoO*_x_*. As one can see, at high minority carrier injection, the carrier lifetime of the Si wafer is limited by Auger recombination (the black dashed line).^91^ Importantly, the thermal evaporation of MoO*_x_* only minimally affects the lifetime of the passivated wafer, as seen from Figure 20.

In contrast, low‐temperature ALD of MoO*_x_* and sputtering of TCO significantly degrade the carrier lifetime. This has been proved to be related to the plasma luminescence and bombardment with high energy particles, which are both absent during thermal evaporation. However, a low‐temperature annealing treatment can almost completely eliminate the detrimental effects on *V*_oc_ from sputtering. From the lifetime measurements, the prediction of an implied‐*V*_oc_ as high as 725 mV at 1 sun confirms the high quality passivation.

### 3.5 High‐Performance TMOs/n‐Si HSCs

#### 3.5.1 Double‐Sided Junctions

**Figure 21:**
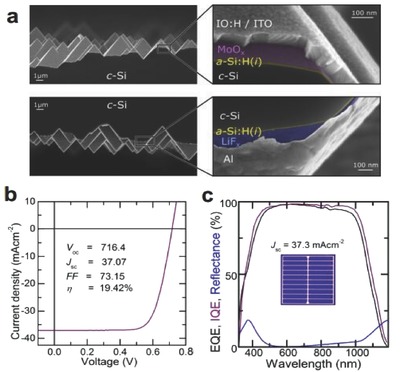
a) Micrometer (LHS)‐ and 100 nm (RHS)‐scale cross‐sectional scanning electron microscopy images of the textured front (sunward side) and back surfaces of the DASH cell. The 100 nm scale image colors are inverted to highlight the different films on each surface; b) light *J–V* behavior and cell characteristics of the DASH cell measured under standard 1‐sun conditions; c) *EQE* (black) and *IQE* (purple) alongside the measured reflectance (blue) for the DASH cells. The *J*_sc_ obtained from the *EQE*, shown above a photograph of the DASH cell, agrees well with that measured from the light *J–V* analysis. Reproduced with permission.^17^ Copyright 2016, Nature Publishing Group.

Bullock et al. first fabricated dopant‐free asymmetric heterocontacts (DASH) implementing the MoO*_x_*/a‐Si:H(i)/n‐Si/a‐Si:H(i)/LiF*_x_* structure, and they obtained an optimized efficiency approaching 20%, as shown in **Figure** **21**.^17^ In their devices, high‐WF MoO*_x_* (≈5.7 eV) was selected as the hole‐selective layer, while an LiF*_x_*/Al contact with a low WF value of ≈2.87 eV and low resistivity value of ≈1 mΩ cm^2^ was designed as an efficient ETL. The insertion of a‐Si:H(i) films greatly suppresses the c‐Si surface recombination rates, allowing high *V*_oc_ values up to 716 mV. For comparison, the n‐ and p‐type DASH cells without a‐Si:H(i) passivating interlayers only provide efficiencies of 11.3% and 13.4%, respectively, proving the importance of interface passivation. An enhancement in the back‐side reflection and a reduction in the series resistance of the DASH cells are identified as the two most likely paths toward even higher efficiency for this design. As a result, an improvement in the *J*_sc_ of ≈1 mA cm^−2^ could arise by replacing Al with Ag or possibly ITO, and a boost in *FF* above 79% could occur by further reducing the resistive losses.^101^, ^102^

#### 3.5.2 IBC Cells Based on TMOs/n‐Si CSCs

The ultimate aim in developing TMOs/n‐Si heterocontacts is to construct IBC‐structured HSCs using an extremely simplified procedure, which is possible because of their dopant‐free characters and easy‐to pattern at lower temperature. With a very low temperature fabrication procedure, the Ni/V_2_O*_x_* stack was used to form the n‐Si IBC HSC architecture. This Ni/V_2_O*_x_*‐based IBC cell resulted in *V*_oc_, *J*_sc_, and an *FF* values of 656 mV, 40.7 mA cm^−2^, and 74.0%, respectively, leading to a *PCE* as high as 19.7%.^98^

**Figure 22:**
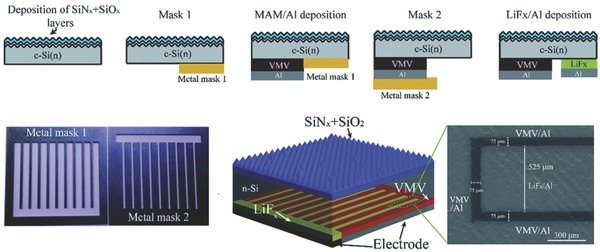
Top) Schematic illustrating the MLBC HSC patterning fabrication process; below 1) images of two metal patterning masks; below 2) the structure of a completed MLBC HSC; below 3) SEM image of the results of metal mask patterning. Reproduced with permission.^82^ Copyright 2017, The Royal Society of Chemistry.

Shen and co‐workers investigated the electrical properties of typical TMO‐based emitters in dopant‐free n‐Si IBC HSCs by comparing the properties of HSCs employing WO*_x_*, MoO*_x_*, and V_2_O*_x_*, as shown in **Figure** **22**.^82^ Among these TMO/n‐Si contacts, the V_2_O*_x_*/n‐Si contact achieved the lowest surface recombination velocity (*S*_eff_) of 138 cm/s and ρ_c_ of 34 mΩ cm^2^, providing a significant advantage over heavily doped a‐Si:H(p)/n‐Si contacts. The relationship between *S*_eff_ and the equilibrium dark band bending ϕ_0_ induced in n‐Si by the TMO layers was also analyzed. The best device performance was achieved by V_2_O*_x_*/n‐Si HSC because V_2_O*_x_* induced the largest quasi‐Fermi‐level splitting within the n‐Si. Novel multilayer back contact (MLBC) HSCs employing V_2_O*_x_* (8 nm)/metal/ V_2_O*_x_* (8 nm) (VMV) multilayers as a dopant‐free hole‐selective contact was also presented using a thermal evaporation process. The lowest ρ_c_ was achieved by using VMV (4 nm Au) as an emitter, leading to an efficiency as high as 19.02% for this type of MLBC HSC. Notably, the author only used two hard masks, rather than photolithographic or other sophisticated masking methods, to complete the whole rear‐sided processes, showing huge potential for the simplification of the procedure over traditional doping techniques.^82^, ^83^

### 3.6 Stability of TMO/Si HSCs

**Figure 23:**
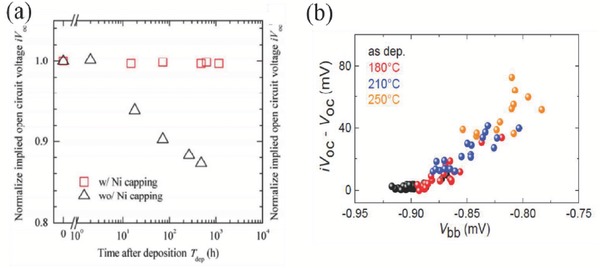
a) Comparative behaviors (time‐dependence of *iV*_oc_ under air exposure) of the V_2_O*_x_*/Si/V_2_O*_x_* samples (40 nm thick V_2_O*_x_*) with and without nickel capping; Reproduced with permission.^98^ Copyright 2017, Royal Society of Chemical. b) dependence of the selectivity of MoO*_x_*/n‐Si heterojunction on the induced c‐Si band bending for different annealing temperatures. Here, *V*_bb_ is the equilibrium band bending, and Δ*V* = *iV*_oc_ − *V*_oc_. Reproduced with permission.^103^ Copyright 2015, Elsevier.

Similar to the PEDOT:PSS/n‐Si heterojunctions, the stability of TMOs/n‐Si contacts must also be investigated and evaluated. As shown in **Figure** **23**a, the *iV*_oc_ for V_2_O*_x_*/Si/V_2_O*_x_* samples (40 nm thick V_2_O*_x_*) degrades with time when exposing V_2_O*_x_* to ambient atmosphere. This might be ascribed to a possible reduction of the material work function by the chemical or adsorption reactions of the samples with air (water and oxygen species), weakening the induced p‐type inversion region and increasing local recombination. Samples with a 10 nm Ni capping layer exhibited a much better long‐term surface passivation stability. However, subjecting as‐fabricated solar cells with Ni and TCO capping layers to annealing at temperatures higher than 130 °C does cause negative effects on the dark *I–V* results and efficiency, which may be caused by interfacial reactions between V_2_O*_x_* and the capping layer.^18^, ^98^ With the contacts of MoO*_x_*/n‐Si, Neusel et al. summarized the effects of post‐annealing treatments on the selectivity of holes, as shown in Figure 23b.^103^ It can be seen that the selectivity is governed by the induced n‐Si band bending. The linear dependence between *V*_bb_ and Δ*V* undoubtedly tell us that the loss of selectivity with annealing is caused by a reduction in this band banding. So, for the TMOs/n‐Si‐based solar cells, the major challenge is to maintain the high WF and favorable interfacial charges (negative) responsible for the induced band bending, regardless of the exposure or annealing treatments.

## 4 Conclusion

In this review, organic PEDOT:PSS and TMOs (i.e., MoO*_x_*, V_2_O*_x_*, and WO*_x_*) have been fully investigated as promising candidates for efficient HTLs to construct dopant‐free and high efficiency Si‐based HSCs. Specifically, progress in research and innovations toward the enhanced performance of PEDOT:PSS/n‐Si HSCs has been comprehensively summarized, including transport mechanisms, PEDOT:PSS modification, work function tuning, advanced design for light‐trapping, interfacial passivation, and improvement of stability. TMOs were also seriously exploited by improving their work function, conductivity, interface passivation quality, and contact resistivity. Moreover, TMOs have been integrated into similar HJT and IBC HSCs, realizing the aim of dopant‐free fabrication methods and high efficiency. Although the current efficiencies of PEDOT:PSS/Si and TMOs/Si HSCs cannot contend with traditional high efficiency doped SCs, researchers continue to devote effort to the promising research field of dopant‐free heterocontacts. We hope that the presentation of the advanced methods and materials in this review may help to inspire a series of breakthroughs to further improve the efficiency of these devices and accelerate their practical applications.

## Conflict of Interest

The authors declare no conflict of interest.
